# Association between dietary vitamin E intake and constipation: NHANES 2005–2010

**DOI:** 10.3389/fnut.2024.1426280

**Published:** 2024-08-20

**Authors:** Junfeng Cai, Danqing Li, Ruijun Xie, Xiaoling Yu, Yuning Wu, Feng Sun, Chenxiong Zhang

**Affiliations:** ^1^The First Clinical Medical College, Guangzhou University of Chinese Medicine, Guangzhou, China; ^2^Department of Traditional Chinese Medicine, Sanming First Hospital, Sanming, China; ^3^Department of Proctology, Yubei Hospital of Traditional Chinese Medicine, Chongqing, China

**Keywords:** Vitamin E, constipation, NHANES, dietary intake, population-based study

## Abstract

**Background:**

This investigation aimed to analyze the association between dietary vitamin E intake and constipation prevalence among United States adults.

**Methods:**

Utilizing data from the National Health and Nutrition Examination Survey (NHANES), this cross-sectional study assessed vitamin E intake through 24-h dietary recall and defined constipation based on the Bristol Stool Form Scale (BSFS). Logistic regression models were employed to evaluate the relationship between vitamin E intake and constipation, with results presented as odds ratios (ORs) and 95% confidence intervals (CIs). Stratified analyses were conducted based on covariates such as age, and restricted cubic spline (RCS) models were generated to explore the potential linear or non-linear association.

**Results:**

Individuals experiencing constipation exhibited lower vitamin E intake compared to those without constipation. Weighted multivariate logistic regression models demonstrated a negative correlation between vitamin E intake and constipation risk, even after adjusting for potential confounding variables. Further RCS analysis revealed a statistically significant non-linear inverse relationship between vitamin E intake and constipation risk (*p*-value for non-linearity = 0.0473).

**Conclusion:**

Our findings suggest an independent inverse association between vitamin E intake and constipation prevalence in United States adults. Prospective research is needed to validate these observations.

## 1 Introduction

Constipation, a widespread chronic gastrointestinal issue, presents with a range of debilitating symptoms including difficulty passing stools, infrequent bowel movements, and a persistent feeling of incomplete evacuation. These symptoms significantly diminish patients’ quality of life (1). With a global prevalence estimated between 10.1 and 15.3% among adults (2), constipation disproportionately affects female individuals, exhibiting a higher incidence in women compared to men (3). The impact of constipation extends far beyond physical discomfort. Individuals struggling with constipation often experience psychological challenges, such as anxiety and depression, and may even exhibit cognitive impairments, further exacerbating their reduced quality of life (4). The Rome Criteria, a widely used diagnostic framework for functional gastrointestinal disorders, categorize constipation into distinct subtypes, such as functional constipation (FC), constipation-predominant irritable bowel syndrome (IBS), defecation disorders (DDs), which involve difficulty with the process of defecation, and opioid-induced constipation (OIC), a common side effect of opioid medications (5). Seeking relief from constipation and its associated discomfort, patients often explore dietary interventions, especially when aiming to minimize the side effects of certain medications. Research suggests that certain dietary components, such as soluble fiber and essential trace elements such as selenium, magnesium, and phosphorus (6–8), may play a role in reducing the risk of chronic constipation. Conversely, diets high in saturated fats (1) or characterized by low energy intake (9) have been associated with an elevated risk of developing this condition. A cross-sectional study conducted in Turkey identified several risk factors contributing to constipation, including physical inactivity, insufficient dietary fiber and water intake, advancing age, female sex, and obesity. These findings underscore the multifactorial nature of constipation and the importance of considering various lifestyle and demographic factors when assessing individual risk (10).

While numerous studies have explored the impact of various nutritional factors on constipation, including the aforementioned fiber intake, fat consumption, and certain micronutrients, there remains a notable gap in our understanding of the potential role of specific vitamins in mitigating this condition. For instance, research has established a connection between vitamin D deficiency and an increased risk of constipation (11). However, the relationship between other fat-soluble vitamins, particularly vitamin E, and constipation has received limited attention in the scientific literature.

Vitamin E is a collective term for a group of fat-soluble compounds, initially discovered in 1922, with diverse biological functions and potential health benefits (12). Its primary role lies in its powerful antioxidant properties, effectively scavenging harmful peroxyl radicals and preventing the chain reaction of free radical propagation within tissues, thereby protecting cells from oxidative damage (13). Moreover, vitamin E plays a crucial role in protecting cellular structures from the detrimental effects of oxidative stress and the damaging byproducts of lipid peroxidation, further contributing to overall cellular health and function (14).

The potential mechanisms linking vitamin E to constipation are multifaceted and warrant further investigation. First, vitamin E’s antioxidant properties may help maintain the integrity of the intestinal mucosa, potentially improving overall gut health and function (15). Second, vitamin E has been shown to modulate inflammatory responses in the gastrointestinal tract, which could influence bowel motility and stool consistency (16). In addition, some studies suggest that vitamin E may enhance the production of prostaglandins, which may play a role in regulating intestinal water and electrolyte absorption, potentially affecting stool softness and ease of passage (17, 18).

Given the potential benefits of vitamin E in gut health and its understudied relationship with constipation, there is a clear need for comprehensive research in this area. The National Health and Nutrition Examination Survey (NHANES) database provides an excellent opportunity to investigate this relationship on a large scale. This extensive dataset allows for a thorough examination of the association between vitamin E intake and constipation while accounting for various confounding factors such as age, sex, dietary habits, and overall health status.

Therefore, this study leverages the comprehensive NHANES database to delve into the potential association between vitamin E intake and constipation. By utilizing this robust dataset, we aim to provide valuable insights into the role of vitamin E in gastrointestinal health and potentially identify new strategies for preventing and managing constipation through nutritional interventions.

## 2 Methods

### 2.1 Data source and study population

The National Health and Nutrition Examination Survey (NHANES) serves as a valuable resource for investigating the health and nutritional status of the United States population. As a publicly available research program conducted by the National Center for Health Statistics (CDC), the NHANES offers a rich dataset representing a diverse cross-section of non-institutionalized individuals across the nation. Employing a rigorous sampling methodology, NHANES selects participants through a stratified, multi-stage, probability-cluster design, ensuring that the collected data accurately reflect the demographics and health characteristics of the broader United States population. To account for the complex sampling design, statistical analyses incorporate appropriate weighting adjustments (19). Adhering to strict ethical standards, the NHANES obtains informed consent from all participants, including parental consent for individuals under 18 years of age. The survey protocol has received ethics approval, and all data collection procedures are conducted in accordance with relevant guidelines and regulations. For the purpose of our investigation, we extracted relevant data from the NHANES cross-sectional study conducted between 2005 and 2010. This timeframe encompasses three 2-year cycles (2005–2006, 2007–2008, and 2009–2010), providing a substantial sample size and allowing for the assessment of potential trends over time.

**Figure 1:**
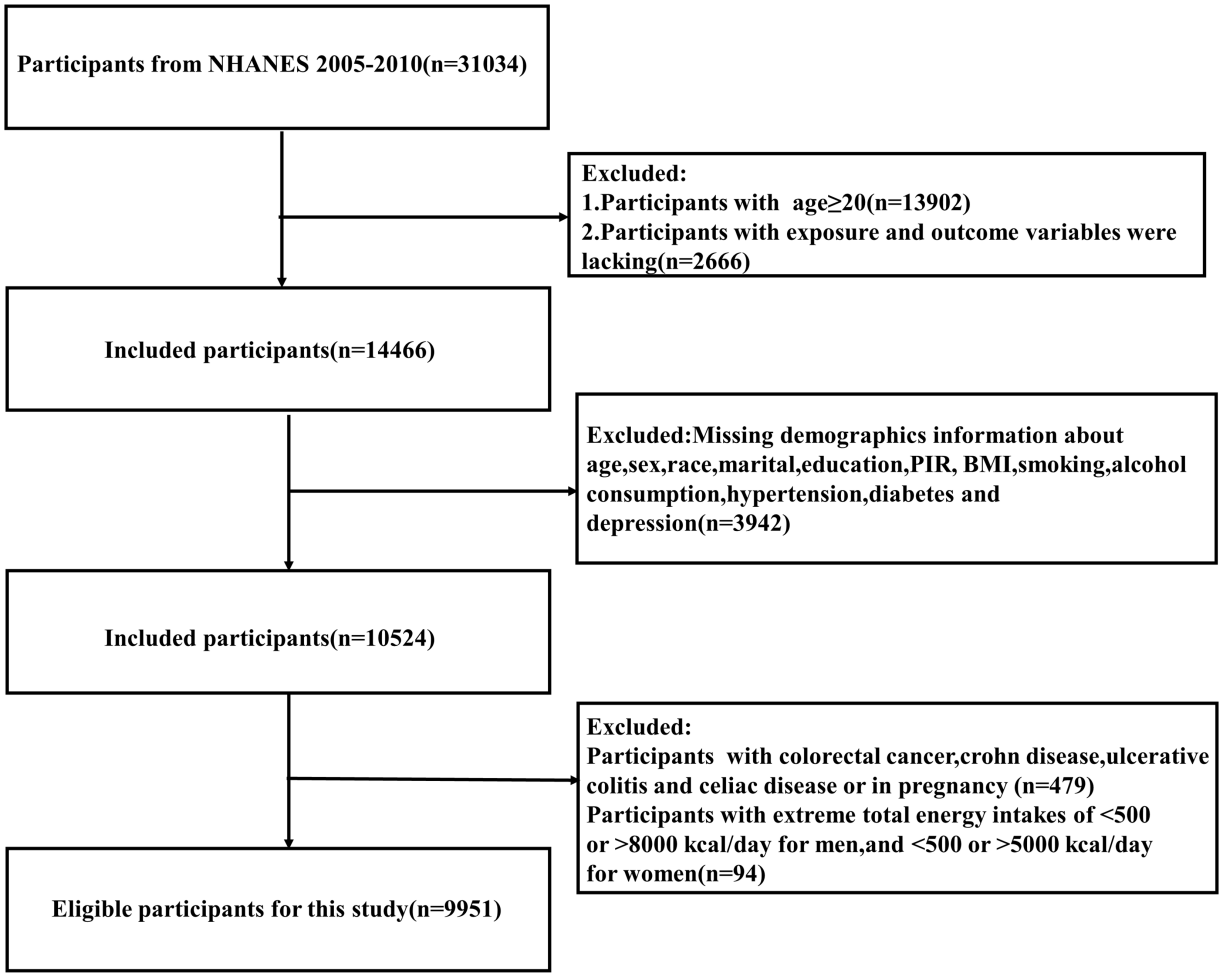
Flowchart of participant screening based on the NHANES database from 2005 to 2010.

The initial data extraction yielded a total of 31,034 participants. To ensure data quality and focus on the relevant adult population for our study, we applied a series of exclusion criteria. We excluded individuals under 20 years of age (*n* = 13,902), participants with missing information on key exposure or outcome variables (*n* = 2,666), and those with incomplete baseline or covariate data (*n* = 3,942), resulting in a remaining sample of 10,524 participants. To refine the study population and minimize potential confounding factors, we further excluded individuals who were pregnant or had a history of colorectal cancer or inflammatory bowel disease (*n* = 479). In addition, to account for potential under- or over-nutrition, we excluded participants with extreme energy intake values based on sex-specific cutoffs (male participants: <500 or > 8,000 kcal/day; female participants: <500 or > 5,000 kcal/day) (*n* = 94) (20). This careful selection process resulted in a final study sample of 9,951 participants, ensuring a focused analysis of the relationship between vitamin E intake and constipation in the general adult population (Figure 1).

### 2.2 Definition of constipation

To assess participants’ bowel habits and identify potential cases of constipation, we utilized data from the common stool type questions included in the NHANES 2005–2010 bowel health questionnaire (21). This standardized questionnaire provides valuable insights into individuals’ bowel movement patterns and characteristics. Data collection for bowel habits during the three NHANES cycles between 2005 and 2010 employed the Bristol Stool Form Scale (BSFS). The BSFS is a widely recognized and validated tool for classifying stool consistency and form. Participants were presented with colored picture cards depicting seven distinct stool types, along with written descriptors, and were asked to identify the image that most closely resembled their usual stool consistency. This approach allowed for a standardized and visual assessment of bowel habits.

The BSFS categorizes stool into seven distinct types, ranging from Type 1 (characterized by separate hard lumps, resembling nuts) to Type 7 (entirely liquid stool with no solid pieces). Types 1 and 2 represent constipation, with stools that are hard, lumpy, and difficult to pass. Types 3 and 4 are considered normal or ideal stool forms, indicating healthy bowel function. Types 5 to 7 represent increasingly loose stools, potentially indicating diarrhea or other digestive issues. Following established criteria for identifying constipation based on stool form, participants who self-reported BSFS Type 1 or 2 stools were classified as experiencing constipation in our study (22, 23). This approach aligns with common clinical practice and allows for a clear distinction between individuals with and without constipation for the purpose of analysis.

### 2.3 Vitamin E intake assessment

The collection and processing of dietary intake data for NHANES participants involved a collaborative effort between NHANES, the United States Department of Agriculture (USDA), and the United States Department of Health & Human Services. This collaboration ensured the accuracy and reliability of the dietary information gathered from participants. To assess vitamin E intake, participants completed two 24-h dietary recall interviews. The first interview was conducted in person at a Mobile Exam Center (MEC), where trained interviewers used standardized protocols to collect detailed information about all foods and beverages consumed by participants during the previous 24-h period. A second interview, conducted by telephone 3 to 10 days later, aimed to capture additional dietary data and enhance the accuracy of intake estimates. It is important to note that NHANES participants did not receive standardized training in food portion size estimation. Given the potential for measurement errors and recall bias associated with 24-h dietary recall, particularly for the second interview conducted remotely, we opted to utilize dietary data solely from the first in-person interview for our analysis (24). This approach prioritized the accuracy and reliability of the dietary intake data used in our study. Our analysis focused specifically on dietary vitamin E intake obtained from food sources and did not account for any vitamin E intake from dietary supplements.

### 2.4 Covariates

To account for potential confounding variables and obtain more accurate estimates of the association between vitamin E intake and constipation, our study incorporated a comprehensive range of covariates reflecting demographic, socioeconomic, lifestyle, and health-related factors. These covariates included age, sex, race, marital status, body mass index (BMI), alcohol consumption, smoking status, education level, family poverty income ratio, and physical activity. Sex was categorized into two groups (male and female); race was classified as non-Hispanic white, Mexican American, non-Hispanic Black, other Hispanic, and other; marital status was divided into widowed/divorced/separated, never married, and married/living with a partner; education level included less than high school (completed grade 9 or less than 9–11 grades), high school or equivalent (high school graduate/GED or equivalent), and more than high school (some college or associate degree and college graduate or above). To assess the impact of socioeconomic status, we categorized the family poverty income ratio, a measure of household income relative to the federal poverty threshold, into three groups: less than 1.3, indicating greater financial hardship; between 1.3 and 3.5, representing moderate income; and greater than 3.5, signifying higher income levels (25). BMI was categorized into four groups: underweight (<18.5), normal (18.5 to <25), overweight (25 to <30), and obese (30+). Smoking status was classified as never, former, and current smokers based on whether they had smoked 100 cigarettes in their lifetime: “Never smokers” referred to individuals who had smoked less than 100 cigarettes in their lifetime, “current smokers” were those who currently smoke, and “former smokers” were those who had smoked more than 100 cigarettes in their lifetime but did not currently smoke (26). Alcohol consumption was categorized as follows: never, for individuals who had consumed less than 12 drinks in their lifetime; former, for those who had consumed at least 12 alcoholic drinks in their lifetime but less than 12 drinks in the past year; current, for those who had consumed more than 12 alcoholic drinks in the past year (27). The definition of vigorous physical activity underwent modifications across different survey cycles. During the 2005–2006 cycle, vigorous physical activity was characterized as engaging in any intense activities for a minimum of 10 min that resulted in heavy sweating, or large increases in breathing or heart rate within the preceding 30 days. For the 2007–2010 period, individuals were considered to have participated in vigorous physical activity if they responded ‘yes’ to the following query: ‘Work involves vigorous-intensity activities that cause significant increases in breathing or heart rate, such as carrying or lifting heavy loads, digging, or construction work for at least 10 min continuously’ (28). In addition, health risk factors, including diabetes, hypertension, and depression, were also considered. By including these health factors as covariates, we aimed to obtain a more precise estimation of the independent association between vitamin E intake and constipation.

### 2.5 Statistical analysis

Recognizing the complex sampling design employed by NHANES, we meticulously weighted the data to ensure accurate and representative results. Following NHANES guidelines, we applied appropriate weights, specifically one-third of the 2005–2010 weights, to account for the sampling methodology and obtain unbiased estimates. The normality of quantitative data was tested using the Kolmogorov–Smirnov test. For baseline characterization, continuous variables with normal distributions were expressed as means with standard deviations (SD), whereas continuous variables with skewed distributions were expressed as medians with interquartile ranges (IQRs). Categorical variables are presented as frequencies and percentages. For normally distributed continuous variables, we utilized Student’s *t*-test to compare group means and assess statistically significant differences between groups, and comparisons among the multiple groups were facilitated through the application of analysis of variance (ANOVA). For variables exhibiting skewed distributions, we employed the Wilcoxon rank-sum test, a non-parametric alternative to the *t*-test suitable for non-normal data. Categorical variables were analyzed using the chi-square test, allowing us to examine the association between different categories and identify potential statistically significant differences in proportions. Given the skewed distribution observed in vitamin E intake levels, we applied a natural log transformation to normalize the data. This transformation, resulting in the variable Log vitamin E, allowed for the application of parametric statistical methods that assume normality. For further analysis, we categorized Log vitamin E into quartiles, creating four groups based on intake levels. Prior to the multivariable analyses, we conducted weighted univariate logistic regression analyses for each covariate to assess its individual association with constipation. Weighted logistic regression analysis was applied to investigate the association between vitamin E intake and constipation. The results are presented as odds ratios (ORs) and 95% confidence intervals (CIs). In Model 1, no covariates were adjusted. Model 2 adjusted for age, sex, race, education level, marital status, and income level. Model 3 further adjusted for BMI, depression, diabetes, hypertension, and other confounding factors based on Model 2. In addition, a trend test (p for trend) was conducted by entering quartiles of vitamin E intake as a continuous variable and re-running the corresponding regression models. Subgroup analyses were performed stratified by all confounding factors. The *p*-value for interaction was used to determine whether the stratified effect was significant. Furthermore, restricted cubic spline (RCS) regression with four knots adjusted for all confounding variables was employed to examine the linear/non-linear relationship between vitamin E intake and constipation. If the association was non-linear, the inflection point of vitamin E intake, the value at which a change in the curve is observed, was sought. All statistical analyses and data processing were conducted using R version 4.3.1,^1^ a widely used statistical software package. Following conventional statistical practice, a two-tailed *p*-value less than 0.05 was considered statistically significant.

## 3 Results

### 3.1 Baseline characteristics of participants

This study included a total of 9,951 individuals between the ages of 20 and 85 years. Table 1 presents the characteristics of the stratified study population based on quartiles of vitamin E intake from the 2005–2010 National Health and Nutrition Examination Survey (NHANES). The analysis revealed significant differences between vitamin E intake levels and various factors. Among demographic characteristics, gender and race were significantly correlated with vitamin E intake levels (*p* < 0.05). Vitamin E intake also showed significant differences across other variables including education level, marital status, poverty income ratio, smoking status, alcohol consumption status, diabetes, and depression (p < 0.05).

**Table 1:** Characteristics of the 99,51 participants from the NHANES 2005–2010 study were analyzed based on their serum vitamin E intake levels.

Variables	Overall	Q1	Q2	Q3	Q4	*p*
*N*	9,951	2,480	2,495	2,489	2,487	
Age, mean(SD)	48.56 ± 17.44	48.65 ± 18.26	49.21 ± 17.81	48.58 ± 17.21	47.80 ± 16.40	0.771
Gender, *n* (%)						<0.001
Male	5,153(51.6)	1,043(42.1)	1,169(46.9)	1,326(53.3)	1,593(64.1)	
Female	4,820(48.4)	1,437(57.9)	1,326(53.1)	1,163(46.7)	894(35.9)	
Race, *n* (%)						<0.001
Mexican American	1732 (17.4)	514 (20.7)	454 (18.2)	413 (16.6)	351(14.1)	
Other Hispanic	806 (8.1)	242 (9.8)	200 (8.0)	199 (8.0)	165(6.6)	
Non-Hispanic Black	5,121 (51.5)	1,063(42.9)	1,266(50.7)	1,343(54.0)	1,449 (58.3)	
Non-Hispanic White	1903 (19.1)	550 (22.2)	482 (19.3)	445 (17.9)	426 (17.1)	
Other race	389 (3.9)	111 (4.5)	93(3.7)	89(3.6)	96(3.9)	
Marital status, *n* (%)						**<0.001**
Married	5,356 (53.8)	1,170(47.2)	1,355(53.9)	1,414(56.4)	1,429 (57.5)	
Widowed	706 (7.1)	235(9.5)	204(8.1)	156(6.2)	112 (4.5)	
Divorced	1,080 (10.9)	287(11.6)	267(10.6)	266(10.6)	265 (10.7)	
Separated	308 (3.1)	91(3.7)	86(3.4)	74(3.0)	58 (2.3)	
Never married	1,697 (17.1)	493(19.9)	416(16.6)	393(15.7)	404 (16.2)	
Living with partner	804 (8.1)	204 (8.2)	184(7.3)	202(8.1)	219 (8.8)	
Education level, *n* (%)						<0.001
<9th Grade	1,022 (10.3)	396 (16.0)	282 (11.3)	202(8.1)	142(5.7)	
9th–11th Grade	1,546 (15.5)	479 (19.3)	391 (15.7)	347(13.9)	329(13.2)	
High School Grad/GED or Equivalent	2,380 (23.9)	648 (26.1)	606 (24.3)	579(23.3)	547(22.0)	
Some College or AA degree	2,853 (28.7)	615 (24.8)	726 (29.1)	778(31.3)	734(29.5)	
College Student or above	2,150 (21.6)	342 (13.8)	490 (19.6)	583(23.4)	735(29.6)	
PIR, *n* (%)						<0.001
<1.3	2,778 (27.9)	930 (37.5)	699 (28.0)	622 (25.0)	527 (21.2)	
1.3–3.5	3,783 (38.0)	1,018 (41.0)	971 (38.9)	934 (37.5)	860 (34.6)	
>3.5	3,390 (34.1)	532 (21.5)	825 (33.1)	933 (37.5)	1,100 (44.2)	
Drinking status, *n* (%)						<0.001
Never	1,302 (13.1)	450 (18.1)	362 (14.5)	275 (11.0)	215 (8.6)	
Former	4,729 (47.5)	1,053(42.5)	1,212(48.6)	1,230(49.4)	1,234 (49.6)	
Now	3,920 (39.4)	977 (39.4)	921 (36.9)	984 (39.5)	1,038 (41.7)	
Smoking status, *n* (%)						<0.001
Never	5,210 (52.4)	1,285(51.8)	1,359(54.5)	1,294 (52.0)	1,272 (51.1)	
Former	2,464 (24.8)	521 (21.0)	600 (24.0)	652 (26.2)	691 (27.8)	
Now	2,277 (22.9)	674 (27.2)	536 (21.5)	543 (21.8)	524 (21.1)	
Diabetes, *n* (%)						**0.027**
Yes	1,018 (10.2)	304 (12.3)	265 (10.6)	238 (9.6)	211 (8.5)	
No	8,745 (87.9)	2,135(86.1)	2,187 (87.7)	2,196 (88.2)	2,227 (89.5)	
Borderline	188 (1.9)	41(1.7)	43 (1.7)	55 (2.2)	49 (2.0)	
Hypertension, *n* (%)						0.262
Yes	3,089 (31.0)	814 (32.8)	755 (30.3)	772 (31.0)	748 (30.1)	
No	6,862 (69.0)	1,666 (67.2)	1740 (69.7)	1717 (69.0)	1739 (69.9)	
BMI, *n* (%)						0.159
<18.5	151 (1.5)	52 (2.1)	30 (1.2)	34 (1.4)	35 (1.4)	
18.5–25	2,758 (27.7)	679 (27.4)	679 (27.2)	687 (27.6)	713 (28.7)	
25–30	3,462 (34.8)	838 (33.8)	848 (34.0)	895 (36.0)	881 (35.4)	
>30	3,580 (36.0)	911 (36.7)	938 (37.6)	873 (35.1)	858 (34.5)	
Depression, *n* (%)						**0.002**
Yes	812 (8.2)	256 (10.3)	209 (8.4)	179 (7.2)	168 (6.8)	
No	9,139 (91.8)	2,224(89.7)	2,286(91.6)	2,310(92.8)	2,319 (93.2)	
Vigorous physical activity, *n* (%)						**<0.001**
Yes	2,392 (24.0)	512 (20.6)	543 (21.8)	649 (26.1)	688 (27.7)	
No	7,559 (76.0)	1968(79.4)	1952 (78.2)	1840(73.9)	1799 (72.3)	

Mean ± standard deviation (SD) for continuous variables. Percentages (%) for categorical variables.PIR, the ratio of family income to poverty; BMI, body mass index. Vitamin E intake was classified according to quartiles, Q1 to Q4.

Table 2 displays the characteristics of the participants according to constipation status (constipated vs. non-constipated). Among them, 745 individuals were identified as having constipation. When comparing the characteristics of constipated and non-constipated individuals, we observed several notable differences. The proportion of female participants was significantly higher in the constipation group compared to the non-constipation group (*p* < 0.05). In addition, we observed a statistically significant difference in vitamin E intake between the two groups. Participants with constipation had significantly lower vitamin E intake compared to those without constipation symptoms (*p* < 0.05). The median vitamin E intake for participants with constipation symptoms was 5.30 mg/day (interquartile range: 3.25–7.89 mg/day), while for those without constipation symptoms, it was 6.15 mg/day (interquartile range: 3.99–9.31 mg/day). Due to the skewed distribution of vitamin E intake among participants, a natural log transformation was applied to vitamin E intake for better subsequent analysis. The results indicated that even after the log transformation, a significant difference in vitamin E intake persisted between individuals with and without constipation.

**Table 2:** Baseline characteristics between constipated and non-constipated participants.

Variables	Non-constipation (*N* = 9,206)	Constipation (*N* = 745)	*p*
Age, mean(SD)	48.76 ± 17.37	46.14 ± 18.13	0.006
Gender, *n* (%)			<0.001
Male	4,886(53.1)	245(32.9)	
Female	4,320(46.9)	500(67.1)	
Race, *n* (%)			<0.001
Mexican American	1,597 (17.3)	135 (18.1)	
Other Hispanic	728 (7.9)	78(10.5)	
Non-Hispanic Black	4,789 (52.0)	332(44.6)	
Non-Hispanic White	1727 (18.8)	176 (23.6)	
Other Race	365 (4.0)	24 (3.2)	
Marital status, *n* (%)			0.070
Married	4,987 (54.2)	369 (49.5)	
Widowed	639 (6.9)	67 (9.0)	
Divorced	990 (10.8)	90 (12.1)	
Separated	288 (3.1)	20 (2.7)	
Never married	1,553 (16.9)	144 (19.3)	
Living with partner	749 (8.1)	55 (7.4)	
Education level, *n* (%)			<0.001
<9th Grade	923 (10.0)	99 (13.3)	
9th–11th Grade	1,410 (15.3)	136 (18.3)	
High School Grad/GED or Equivalent	2,168 (23.5)	212 (28.5)	
Some College or AA degree	2,672 (29.0)	181 (24.3)	
College Student or above	2033 (22.1)	117 (15.7)	
PIR, *n* (%)			<0.001
<1.3	2,513 (27.3)	265 (35.6)	
1.3–3.5	3,491 (37.9)	292 (39.2)	
>3.5	3,202 (34.8)	188 (25.2)	
Drinking status, *n* (%)			0.009
Never	1,164 (12.6)	138 (18.5)	
Former	4,375 (47.5)	354 (47.5)	
Now	3,667 (39.8)	253 (34.0)	
Smoking status, *n* (%)			0.002
Never	4,763 (51.7)	447 (60.0)	
Former	2,325 (25.3)	139 (18.7)	
Now	2,118 (23.0)	159 (21.3)	
Diabetes, *n* (%)			0.058
Yes	941 (10.2)	77 (10.3)	
No	8,085 (87.8)	660 (88.6)	
Borderline	180 (2.0)	8 (1.1)	
Hypertension, *n* (%)			0.024
Yes	2,887 (31.4)	202 (27.1)	
No	6,319 (68.6)	543 (72.9)	
BMI, *n* (%)			0.003
<18.5	133 (1.4)	18 (2.4)	
18.5–25	2,509 (27.3)	249 (33.4)	
25–30	3,209 (34.9)	253 (34.0)	
>30	3,355 (36.4)	225 (30.2)	
Depression, *n* (%)			<0.001
Yes	710 (7.7)	102 (13.7)	
No	8,496 (92.3)	643 (86.3)	
Vigorous physical activity, *n* (%)			0.001
Yes	2,250 (24.4)	142 (19.1)	
No	6,956 (75.6)	603 (80.9)	
Vitamin E, median(IQR)	6.15(3.99–9.31)	5.30(3.25–7.89)	<0.001
Log Vitamin E, median(IQR)	1.82(1.38–2.23)	1.67(1.18–2.07)	<0.001

Mean ± standard deviation (SD) for continuous variables, median (interquartile range) for continuous variable that is not normally distributed. Percentages (%) for categorical variables.PIR, the ratio of family income to poverty; BMI, body mass index.

### 3.2 Association between vitamin E intake and the risk of constipation

**Figure 2:**
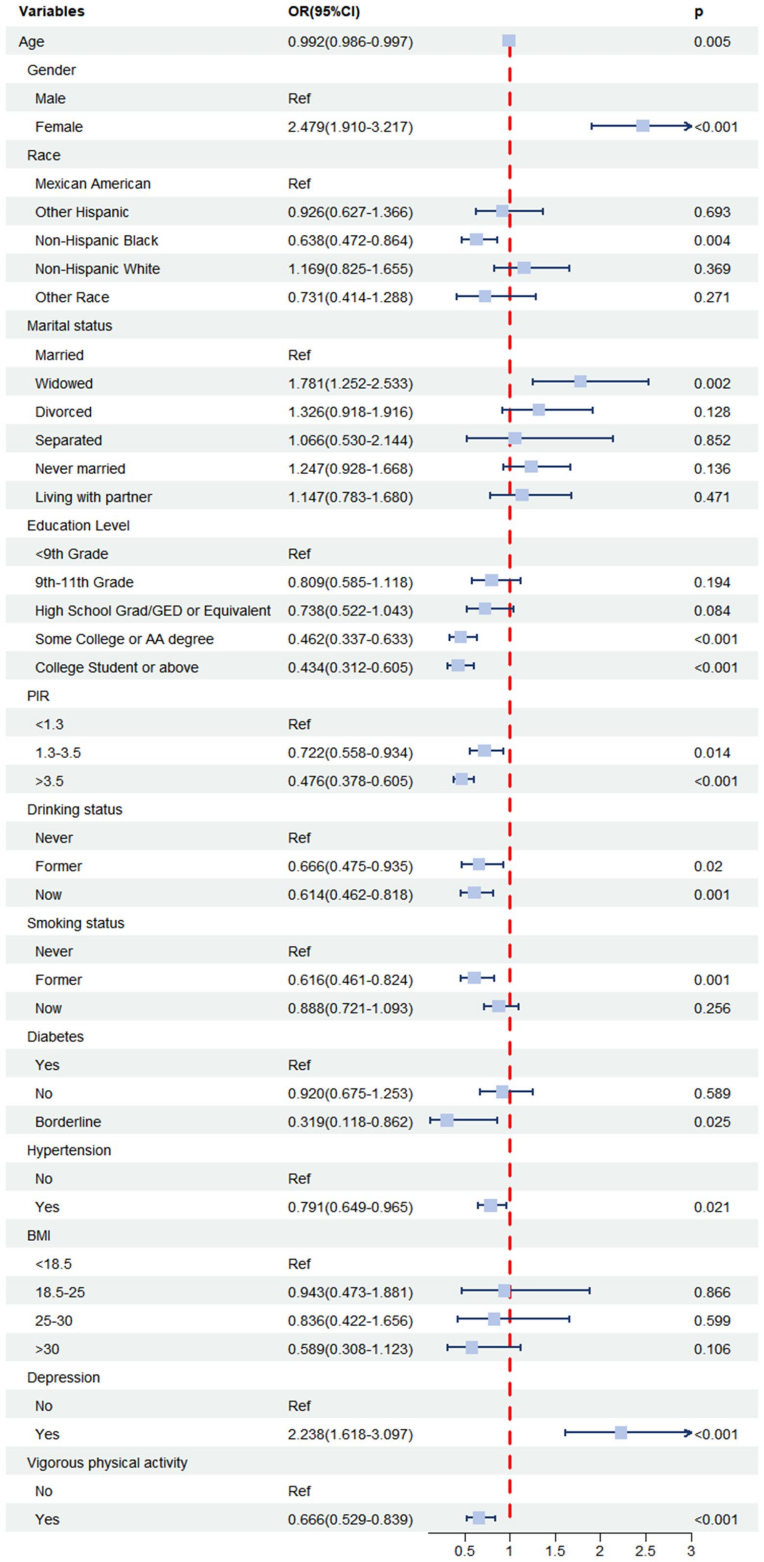
Forest plot displaying the associations between each covariate and constipation.

Prior to the multivariable analyses, we conducted weighted univariate logistic regression analyses for each covariate to assess its association with constipation (Table 3 and Figure 2). We found that female participants had a significantly higher risk of constipation compared to male participants (OR = 2.479, 95% CI: 1.910–3.217, *p* < 0.001), while individuals with higher education levels exhibited a lower risk of constipation compared to those with lower education levels. Similarly, higher income was associated with a lower risk of constipation compared to lower income. In addition, individuals with depression had a significantly higher risk of constipation compared to those without depression (OR = 2.238, 95% CI: 1.618–3.097, *p* < 0.001).

**Table 3:** Single factor weighted logistic regression analysis.

Variables	Univariate OR (95%CI)	*p*
Age	0.992 (0.986–0.997)	0.005
Gender		
Male	Ref	
Female	2.479 (1.910–3.217)	<0.001
Race		
Mexican American	Ref	
Other Hispanic	0.926 (0.627–1.366)	0.692
Non-Hispanic Black	0.638 (0.472–0.864)	0.004
Non-Hispanic White	1.169 (0.825–1.655)	0.369
Other Race	0.731 (0.414–1.288)	0.271
Marital status		
Married	Ref	
Widowed	1.781 (1.252–2.533)	0.002
Divorced	1.326 (0.918–1.916)	0.128
Separated	1.066 (0.530–2.144)	0.852
Never married	1.247 (0.929–1.672)	0.136
Living with partner	1.147 (0.783–1.680)	0.471
Education level		
<9th Grade	Ref	
9th–11th Grade	0.809 (0.585–1.118)	0.194
High school grad/GED or equivalent	0.738 (0.522–1.043)	0.084
Some college or AA degree	0.462 (0.337–0.633)	<0.001
College student or above	0.434 (0.312–0.605)	<0.001
PIR		
<1.3	Ref	
1.3–3.5	0.722 (0.558–0.934)	0.014
>3.5	0.476 (0.378–0.599)	<0.001
Drinking status		
Never	Ref	
Former	0.659 (0.472–0.921)	0.016
Now	0.614 (0.462–0.815)	0.001
Smoking status		
Never	Ref	
Former	0.616 (0.461–0.824)	0.001
Now	0.888 (0.721–1.093)	0.256
Diabetes		
Yes	Ref	
No	0.920 (0.675–1.253)	0.589
Borderline	0.319 (0.118–0.862)	0.025
Hypertension		
No	Ref	
Yes	0.791 (0.649–0.965)	0.021
BMI		
<18.5	Ref	
18.5–25	0.943 (0.473–1.881)	0.866
25–30	0.836 (0.422–1.656)	0.599
>30	0.589 (0.308–1.123)	0.106
Depression		
No	Ref	
Yes	2.238 (1.618–3.097)	<0.001
Vigorous physical activity		
No	Ref	
Yes	0.666 (0.529–0.839)	<0.001

**Figure 3:**
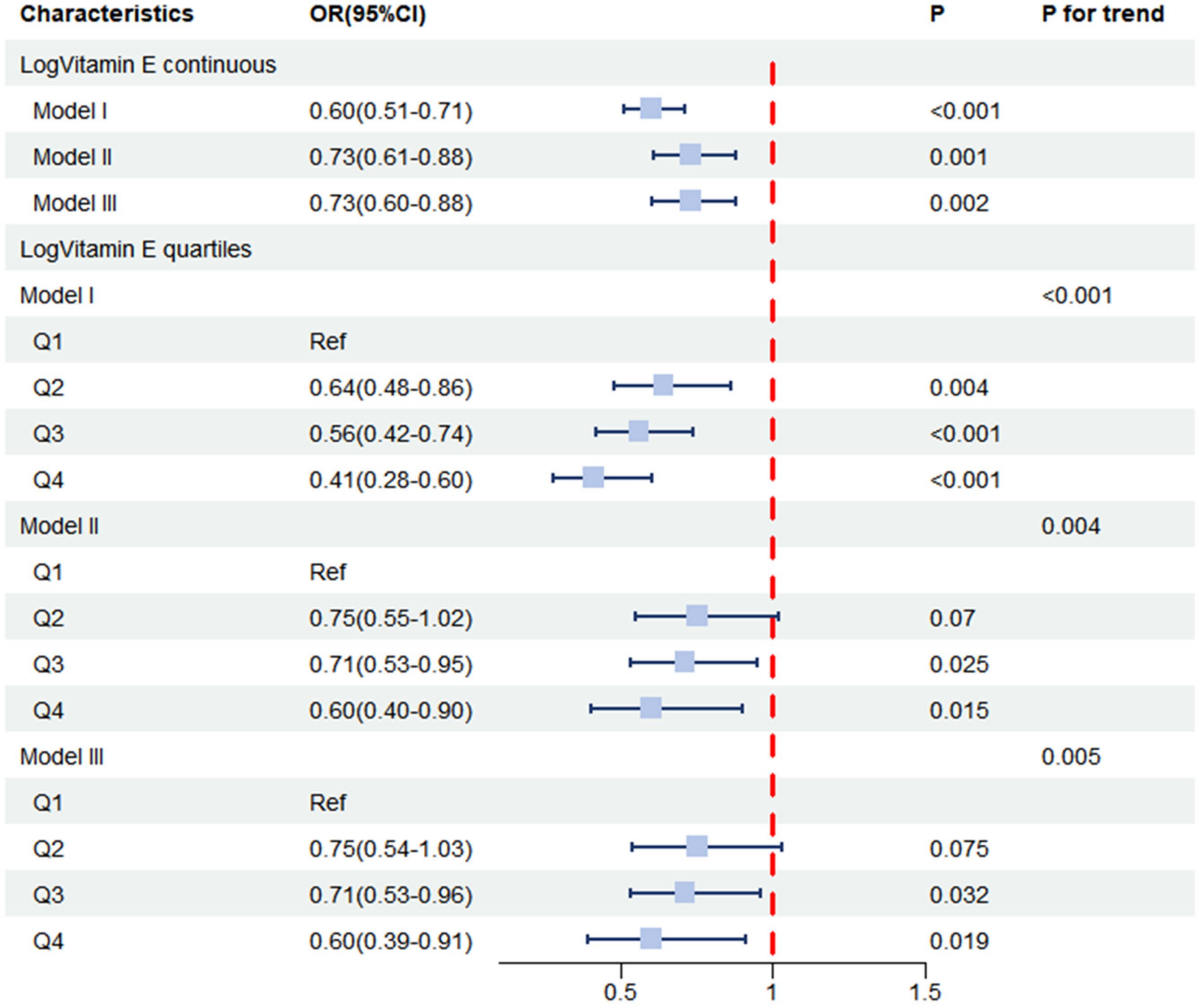
Forest plot displaying the associations between Log vitamin E intake and constipation.

We then performed multivariable logistic regression analyses (Table 4 and Figure 3), where Model 1 was unadjusted for any factors, while Model II was adjusted for basic characteristics including age, race, sex, marital status, income level, and education level. Model III was further adjusted for all potential confounders. The results of the weighted logistic regression analyses indicated that higher vitamin E levels were associated with a lower risk of constipation. When examining the continuous form of Log Vitamin E, Model I, which did not adjust for any covariates, revealed a strong negative association between vitamin E intake and constipation risk (OR = 0.60, 95% CI: 0.51–0.71, *p* < 0.001). This association persisted even after adjusting for demographic and socioeconomic factors in Model II (OR = 0.73, 95% CI: 0.61–0.88, *p* = 0.001) and remained robust after further adjustment for all potential confounders in Model III (OR = 0.73, 95% CI: 0.60–0.88, *p* = 0.002). These findings highlight the independent nature of the association between vitamin E intake and constipation risk, suggesting that it is not solely explained by other factors considered in the analysis. Based on the results from Model III, we estimated that for each unit increase in Log Vitamin E intake, the odds of experiencing constipation decreased by 27%, providing a quantifiable measure of the protective effect associated with higher vitamin E intake.

**Table 4:** Weighted logistic regression of the association between log Vitamin E intake and constipation.

	Model I		Model II		Model III	
	OR (95%CI)	*P*- value	OR (95%CI)	*P*- value	OR (95%CI)	*P*- value
Log Vitamin E	0.60 (0.51–0.71)	<0.001	0.73 (0.61–0.88)	0.001	0.73 (0.60–0.88)	0.002
Log Vitamin E quartiles						
Q1	Ref		Ref		**Ref**	
Q2	0.65 (0.48–0.87)	0.005	0.75 (0.55–1.02)	0.086	0.75 (0.55–1.04)	0.075
Q3	0.56 (0.42–0.75)	<0.001	0.71 (0.53–0.95)	0.030	0.72 (0.53–0.97)	0.032
Q4	0.42 (0.29–0.61)	<0.001	0.60 (0.40–0.90)	0.018	0.60 (0.40–0.91)	0.019
P for trend		<0.001		0.004		0.005

Model I: adjusted for no covariates.Model II: adjusted for age, sex, race, marital status, education level, and PIR.Model III: adjusted for all covariates.

For sensitivity analysis, we stratified Log vitamin E from continuous form into quartile form. From the fully adjusted model, the group in the highest quartile of Log vitamin E exhibited a lower risk of constipation compared to the group in the lowest quartile (OR = 0.60, 95% CI: 0.39–0.91, *p* = 0.019). Similar results were observed in the unadjusted model (Model I) and the partially adjusted model (Model II). Trend analysis revealed a decreasing trend in the association between Log vitamin E and constipation (*P* for trend <0.001 in the unadjusted model, *P* for trend = 0.004 in Model II, and *P* for trend = 0.005 in Model III).

### 3.3 Subgroup analysis

**Figure 4:**
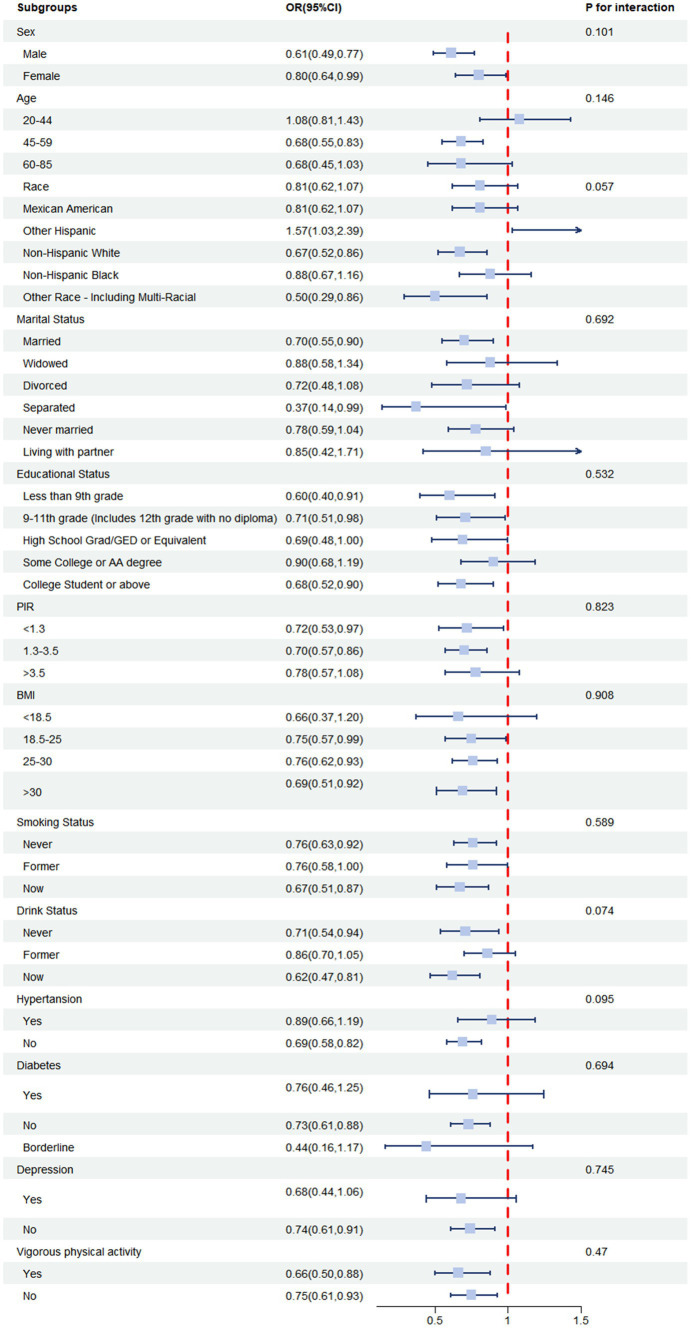
ORs (95% CI) from multiple logistic regression analysis models of associations between Log vitamin E intake and risk of constipation in different subgroups.

To assess the potential effect of modification of Log vitamin E intake on constipation, stratified analyses were conducted, including various subgroups. The results of these analyses are presented in the forest plot in Figure 4. The results revealed that the association between Log vitamin E intake and constipation risk was generally significant across multiple subgroups. In the interaction analysis, we examined potential differences in the association between Log vitamin E intake and constipation risk across various subgroups. Our comprehensive analysis did not reveal any statistically significant interactions (all *p*-values for interaction >0.05). This suggests that the relationship between vitamin E intake and constipation risk is relatively consistent across the different groups in our study population.

### 3.4 Dose–response relationship between log vitamin E intake and risk of constipation

**Figure 5:**
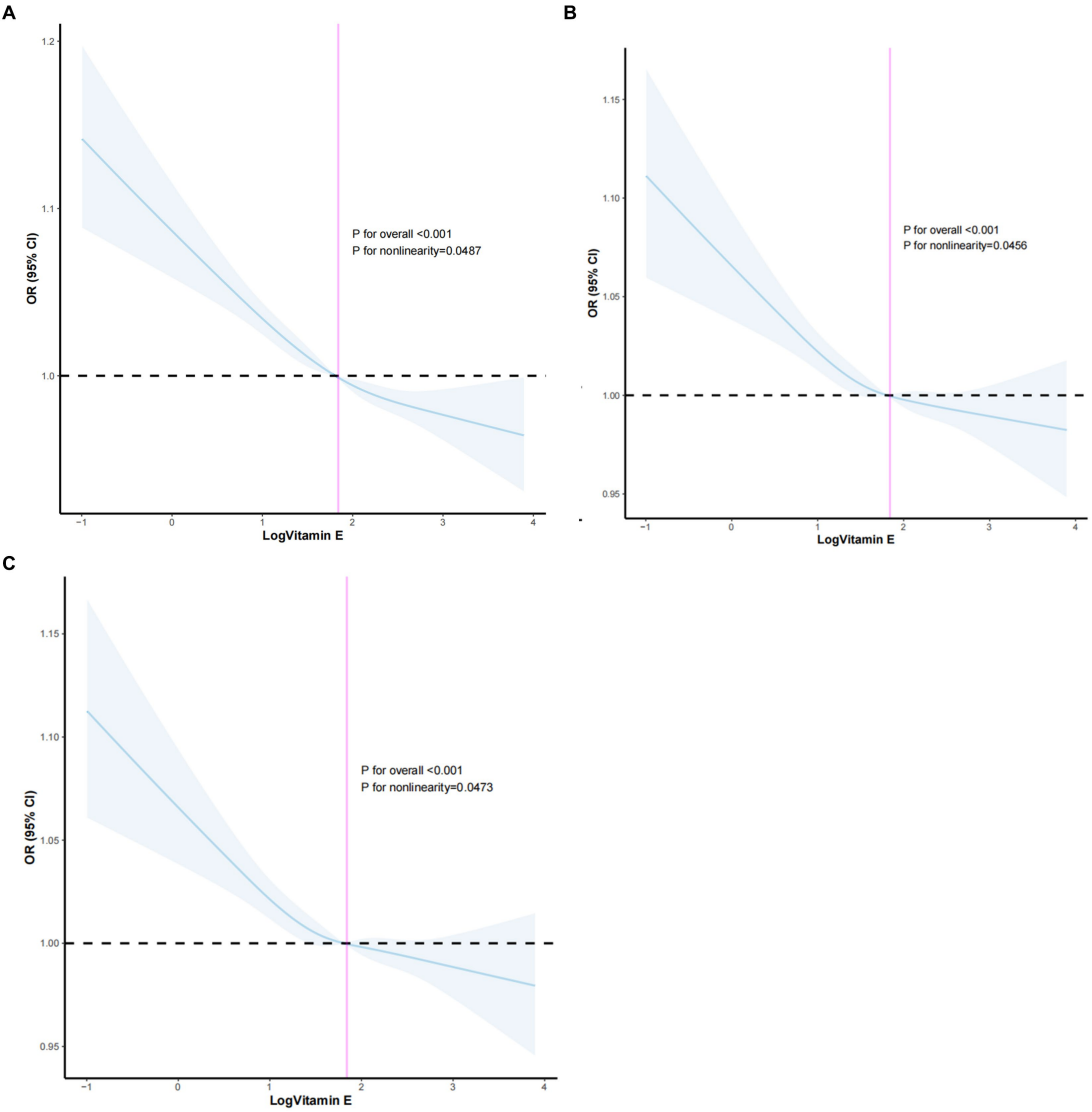
**(A)** RCS plot adjusted for no covariates. **(B)** RCS plot adjusted for age, sex, race, marital status, education level, and PIR. **(C)** RCS plot adjusted for all covariates.

To gain a deeper understanding of the nature of the association between Log vitamin E intake and constipation risk, we employed restricted cubic spline (RCS) analysis. This flexible statistical technique allowed us to visually explore the potential for both linear and non-linear relationships while accounting for the influence of confounding variables. We utilized four knots in the RCS model, strategically placed to capture potential changes in the direction or magnitude of the association across the range of Log vitamin E intake values (Figure 5). The RCS curve visually depicted a clear and consistent pattern, demonstrating a steady decrease in constipation risk as Log vitamin E intake increased. In the unadjusted RCS model, the *p*-value for the non-linear relationship was less than 0.05, with an overall *p*-value <0.001, suggesting a non-linear relationship between Log vitamin E intake and constipation risk as statistically significant evidence of non-linearity was found. Similar conclusions were drawn in Model II, with partial adjustment for confounders, and Model III, with full adjustment for confounders, enhancing the robustness of the results.

## 4 Discussion

To our knowledge, this investigation represents the pioneering exploration of the association between vitamin E intake and constipation within the adult population. We established inclusion and exclusion criteria and ultimately included a total of 9,951 United States adults to investigate the association between vitamin E intake and constipation. Participants with constipation had lower vitamin E intake levels compared to non-constipated participants. Lower vitamin E intake levels were associated with a higher risk of constipation. Subgroup analyses did not reveal any potential interactions between the variables of subgroups and the association between vitamin E intake and constipation risk. This may suggest that the relationship between vitamin E intake and constipation risk is relatively consistent across different demographic groups in our study population. In addition, RCS curve analysis indicated a non-linear relationship between Log vitamin E intake levels and constipation risk. Therefore, supplementation of vitamin E intake appears to significantly contribute to the prevention of constipation. In terms of demographic characteristics, our findings indicated that female participants (29), individuals with normal or lower BMI (30), and those with low-to-middle income levels (31) tend to have a higher prevalence of constipation. Our study also found that individuals engaging in vigorous physical activity had a lower incidence of constipation (28), which aligns with previous research findings.

Vitamin E plays a crucial role in human health, acting as an essential fat-soluble nutrient with diverse biological functions. As it cannot be synthesized by the human body, vitamin E must be obtained through dietary sources. Fortunately, vitamin E is abundant in various foods, including nuts, seeds, vegetable oils (such as coconut, maize, palm, and olive oils), green leafy vegetables, and fortified food products (32). Vitamin E deficiency is generally considered rare. According to the Office of Dietary Supplements of the National Institutes of Health (33), the recommended daily dietary intake for adults is 15 mg of α-tocopherol (equivalent to 22.4 or 33.3 IU of natural or synthetic α-tocopherol, respectively). The relationship between constipation and vitamin E is not yet fully understood. A case–control study conducted in Malaysia on the dietary patterns of IBS-C patients found that dietary vitamin E intake was significantly lower in IBS-C patients compared to healthy individuals (34). Zhou et al. conducted a randomized controlled trial with 70 children with chronic constipation and 70 age- and sex-matched healthy children, which included measuring plasma vitamin E levels. The results showed that plasma vitamin E levels in children with chronic constipation were significantly lower than those in healthy children (35). These findings are consistent with the conclusions of this article, further validating our research hypothesis.

However, the biological mechanisms underlying this potential association remain unclear. A review suggests that the generation of reactive oxygen species (ROS) and oxidative stress plays a crucial role in the pathogenesis and progression of many diseases, including intestinal diseases. Excessive ROS can lead to damage to cellular structures and molecules such as lipids, proteins, and DNA, ultimately resulting in intestinal diseases (36–38). Interstitial cells of Cajal (ICCs) play a critical role in regulating intestinal motility by acting as pacemaker cells within the intestinal smooth muscle. Oxidative stress can disrupt ICC function and promote apoptosis (cell death), leading to impaired intestinal motility and potentially contributing to the development of constipation (39). In a mouse model of postoperative intestinal obstruction, surgical manipulation can lead to increased oxidative stress levels in intestinal tissues. Oxidative stress can damage intestinal tissues, including mucosa and muscularis, leading to intestinal motility dysfunction. Moreover, oxidative stress can activate inflammatory signaling pathways such as the MAPK signaling pathway, promoting the release of inflammatory mediators and infiltration of leukocytes, exacerbating intestinal inflammation and further aggravating intestinal obstruction (40). Several studies have shown that children with chronic constipation have underlying oxidative stress, manifested by decreased antioxidant levels and increased lipid peroxidation levels. This oxidative imbalance may be associated with intestinal dysfunction and increased toxin absorption and may exacerbate constipation symptoms (35, 41). As an important antioxidant, vitamin E plays a crucial role in protecting cells from oxidative damage. It effectively scavenges harmful free radicals, neutralizing their damaging effects and preventing oxidative stress-induced cellular injury (42–44). Given the established role of oxidative stress in constipation and the antioxidant properties of vitamin E, we hypothesize that vitamin E may contribute to alleviating constipation symptoms by mitigating oxidative stress within intestinal tissues, thereby protecting the intestinal barrier and promoting healthy bowel motility. This potential mechanism warrants further investigation to fully elucidate the protective effects of vitamin E in the context of constipation.

Beyond its antioxidant capacity, vitamin E may also exert beneficial effects on gut health by influencing the composition and function of the gut microbiota. Studies suggest that vitamin E can promote the growth of beneficial bacteria within the gut, enhance the production of short-chain fatty acids (SCFAs), and strengthen the intestinal barrier, collectively contributing to improved digestive health (45, 46). The gut microbiota plays a vital role in influencing intestinal motility. Some potential mechanisms include the following: the gut microbiota and its metabolites (such as short-chain fatty acids and tryptophan metabolites) influence enteric nervous system function through Toll-like receptors and serotonin signaling pathways, thereby regulating intestinal motility (47). Certain bacteria are involved in the deconjugation and dehydroxylation of primary bile acids, producing secondary bile acids. Secondary bile acids can activate TGR5 receptors, regulating intestinal motility and secretion (48). Dysbiosis, an imbalance in the gut microbiota composition, has been observed in individuals with constipation. Studies have reported a decrease in the abundance of *Prevotella* genus and an increase in certain *Firmicutes* genera within the gut microbiota of constipation patients compared to healthy controls (49).

Based on the collective evidence from our study and existing research, we propose that vitamin E may hold promise in alleviating constipation symptoms through its antioxidant properties and its ability to modulate the gut microbiota. To substantiate these hypotheses and establish a more definitive link between vitamin E and constipation management, further research, including well-designed intervention studies and mechanistic investigations, is warranted. These future studies will contribute valuable insights into the therapeutic potential of vitamin E for constipation and guide evidence-based recommendations for dietary and supplementation strategies.

In summary, to our knowledge, our study is the first to investigate the relationship between vitamin E intake and constipation in the entire adult population (from young adults to older adults). This study has several strengths. First, the study population came from the NHANES study, with a nationally representative sample, providing a sufficient basis for the research findings. Second, we considered various potential confounders to better estimate the association between vitamin E intake and constipation. However, it is crucial to acknowledge several important limitations of this study. First and foremost, the cross-sectional nature of this study precludes any causal inference regarding the relationship between vitamin E intake and constipation. While we observed an association, we cannot determine whether increased vitamin E intake leads to reduced constipation or if individuals with less constipation tend to have diets higher in vitamin E. This limitation is inherent to cross-sectional studies and highlights the need for longitudinal research to establish any potential causal relationship (50). Second, our reliance on dietary recalls may not accurately capture the participants’ usual vitamin E intake. While this method is commonly used in large-scale nutritional studies due to its feasibility, it has limitations in representing long-term dietary patterns. Day-to-day variations in diet and potential recall bias could lead to misclassification of vitamin E intake levels. This limitation may affect the precision of our estimates and potentially attenuate the observed associations. Future studies could benefit from using more comprehensive dietary assessment methods, such as food frequency questionnaires or multiple 24-h recalls over an extended period, to better represent habitual vitamin E intake.

Third, random measurement error is inevitable in dietary recall data and may bias the results. This measurement error could potentially lead to an underestimation of the true association between vitamin E intake and constipation. Finally, due to differences in genetic background, metabolism, and vitamin E intake levels, the United States findings may not be generalizable to other populations. This limits the external validity of our results and underscores the need for similar studies in diverse populations. Despite these limitations, our study provides valuable initial insights into the potential relationship between vitamin E intake and constipation. To address these limitations, future research should prioritize longitudinal study designs to establish causality, employ more comprehensive dietary assessment methods to accurately capture long-term vitamin E intake, and investigate this relationship in diverse populations.

## 5 Conclusion

In this study, we observed a negative association between vitamin E intake and constipation among United States adults. This may suggest a potential role for vitamin E in the prevention and management of constipation. Further research is warranted to confirm the potential benefits of adequate vitamin E intake in mitigating constipation and elucidate the underlying mechanisms.

## Data Availability

Publicly available datasets were analyzed in this study. This data can be found at: https://www.cdc.gov/nchs/nhanes/index.htm.

## Footnotes

^1^ https://www.r-project.org/
